# Beyond Glycated Hemoglobin: Changes in Time in Range Following Structured Carbohydrate-Counting Education in Adults with Type 1 Diabetes

**DOI:** 10.3390/nu18213640

**Published:** 2026-10-31

**Authors:** Nevin Avhan, Gözde Arıtıcı Çolak, Murat Baş

**Affiliations:** 1 Department of Nutrition and Dietetics, Graduate School of Health Sciences, Acibadem Mehmet Ali Aydinlar University, 34752 Istanbul, Turkey; 2 Department of Nutrition and Dietetics, Faculty of Health Sciences, Acibadem Mehmet Ali Aydinlar University, 34752 Istanbul, Turkey

**Keywords:** carbohydrate counting, type 1 diabetes, time in range, continuous glucose monitoring, glycemic variability, medical nutrition therapy, insulin-to-carbohydrate ratio

## Abstract

**Background/Objectives:** Carbohydrate counting is recommended for adults with type 1 diabetes using flexible prandial insulin, but its association with continuous glucose monitoring (CGM) metrics in adults already using CGM is less well characterized. We evaluated changes in time in range (TIR), glycemic variability, and glycated hemoglobin (HbA1c) following dietitian-led structured carbohydrate-counting education. **Methods:** In this 12-week single-arm pre–post study, 38 adults with type 1 diabetes using CGM were enrolled in an eight-visit, three-level education program and 30 completed the 12-week follow-up; all analyses are completers analyses restricted to these 30 participants. Fourteen-day CGM metrics, HbA1c, insulin doses, anthropometric measures, and dietary intake were assessed at baseline and week 12. TIR (70–180 mg/dL) was designated the primary outcome after data collection and analysis, so this designation is post hoc. **Results:** TIR increased from 62.5% ± 14.9% to 73.0% ± 10.9% (mean difference +10.4 percentage points, 95% CI 7.1 to 13.8; *p* < 0.001). Time above range decreased at 181–250 mg/dL (*p* = 0.002) and >250 mg/dL (*p* < 0.001), and HbA1c decreased from 7.71% ± 1.17% to 7.08% ± 0.57% (*p* < 0.001), without an increase in time below range. The coefficient of variation was lower at week 12 than at baseline (35.7% to 33.6%; *p* = 0.029), although mean baseline variability was already below the recommended 36% threshold; this difference was not statistically significant in a sensitivity analysis restricted to participants meeting the body mass index eligibility criterion (*n* = 22; *p* = 0.179). No increase in body weight or total daily insulin dose was detected. **Conclusions:** In this uncontrolled study, structured carbohydrate-counting education was associated with higher TIR and lower HbA1c without an increase in time below range. These findings are hypothesis-generating and require confirmation in controlled studies.

## 1. Introduction

Type 1 diabetes stems from immune-mediated destruction of pancreatic beta cells, necessitating lifelong exogenous insulin therapy [1]. Medical nutrition therapy represents an integral component of diabetes management, particularly for individuals receiving flexible prandial insulin dosing [2]. Current guidelines highlight personalized medical nutrition therapy and education on the glycemic effects of carbohydrate, fat, and protein, tailored to the individual’s insulin plan and needs to optimize mealtime insulin dosing [3].

Carbohydrate counting is based on the association between the amount of carbohydrates consumed at a meal and the prandial insulin required for managing the resulting postprandial glucose response [4]. The Diabetes Control and Complications Trial systematically incorporated the role of carbohydrate counting in intensive diabetes management into the nutrition intervention [5]. Structured carbohydrate-counting education can be progressively delivered, from identifying carbohydrate-containing foods and estimating carbohydrate intake to applying individualized insulin-to-carbohydrate ratios (ICRs) for prandial insulin adjustment [6]. Systematic reviews and meta-analyses have reported improvements in glycemic control following carbohydrate-counting interventions; however, the magnitude and consistency of these effects have differed across studies [7,8].

However, in clinical practice, carbohydrate counting may be reduced to formula-based estimation rather than delivered as a structured educational process. Conventional approaches estimate the ICR from total daily insulin dose; however, formula-derived estimates may vary from individually determined ratios, and insulin requirements may be inconsistent across meals [9,10]. Moreover, an association exists between lower carbohydrate-counting accuracy and greater glucose variability [11]. These observations underscore the distinction between applying a numerical formula and developing the skills essential for more accurately managing carbohydrate intake and prandial insulin.

Continuous glucose monitoring (CGM) has expanded glycemic management assessment beyond glycated hemoglobin (HbA1c) by providing information on glucose exposure and variability [12]. Time in range (TIR; 70–180 mg/dL), time above range (TAR), time below range (TBR), and coefficient of variation (CV) offer complementary information not captured by HbA1c alone. International consensus recommendations established standardized CGM targets [13]; current clinical standards continue to incorporate these metrics into glycemic assessment, including a TIR goal of >70% for most nonpregnant adults using CGM [14].

Recent evidence has begun extending the evaluation of carbohydrate-counting interventions from HbA1c to CGM-derived outcomes. A 2025 systematic review and meta-analysis of telemedicine-delivered carbohydrate-counting interventions revealed HbA1c and TIR improvements [15]. Similarly, recent clinical studies have reported promising glycemic outcomes following structured nutrition education accompanying glucose-sensor initiation [16] and technology-supported carbohydrate-counting and bolus-calculation interventions [17].

However, recent studies have mainly evaluated telemedicine-based interventions, nutrition education concurrently delivered with glucose-sensor initiation, or application-based carbohydrate-counting and bolus-calculation support [15,16,17]. Face-to-face structured education itself is well established: the Dose Adjustment for Normal Eating (DAFNE) trial demonstrated that a 5-day structured skills program improved both glycemic control and quality of life in adults with type 1 diabetes [18], and comparable structured programs have since been delivered in several countries. These pivotal trials, however, preceded routine CGM use and reported HbA1c rather than CGM-derived metrics. What remains less well characterized is how a progressive, individually delivered dietitian-led carbohydrate-counting program relates to CGM-derived metrics in adults who are already using CGM. Therefore, this study evaluated changes in TIR, glycemic variability, and HbA1c following a diabetes dietitian-led structured three-level carbohydrate-counting education program delivered to adults with type 1 diabetes on CGM. We further investigated whether these changes occurred without an increase in insulin requirements or body weight, or substantial changes in dietary intake, and compared the individually determined ICRs with conventional 500 and 450 rule-derived estimates.

## 2. Materials and Methods

### 2.1. Study Design and Setting

This was a single-arm pre–post interventional study wherein participants served as their own control. This study was conducted at the Turkish Diabetes Foundation Küçükyalı Internal Medicine Center, Istanbul, Turkey. The first participant was enrolled on 8 February 2022, whereas the final participant was enrolled on 21 January 2023; this study was completed on 21 May 2023. Each participant was followed for 12 weeks from the first education visit.

The Clinical Research Ethics Committee of Acıbadem Mehmet Ali Aydınlar University approved the study protocol on 24 March 2021 (decision no. 2021-06/09), before participant enrollment. This study was conducted following the Declaration of Helsinki, and written informed consent was obtained from all participants before enrollment. The study was retrospectively registered with ClinicalTrials.gov (NCT07834801) on 17 September 2026. The study was conducted as an investigator-initiated doctoral thesis project and was not registered prospectively; registration was completed after data collection and before submission of this manuscript, and this is acknowledged as a limitation. The registered primary outcome is the change in TIR (70–180 mg/dL) from baseline to week 12, and the registered secondary outcomes are the changes in CV and laboratory-measured HbA1c; these designations are used in the present report. Because registration followed data collection, the designation of a primary outcome was made after the data had been collected and analyzed and is therefore post hoc rather than prespecified in a public registry. The ClinicalTrials.gov record was first submitted and first posted on 17 September 2026, after data collection had been completed (May 2023) and the analysis for the doctoral thesis had been performed. The ethics-approved protocol listed CGM-derived glycemic variability markers (including TIR and CV), insulin dose, and cardiometabolic risk factors as outcomes without designating a primary outcome; lipid profile and blood pressure were not available for a sufficient number of participants and are not reported here.

### 2.2. Participants

Adults aged 19–65 years with type 1 diabetes for at least 1 year were eligible when they were receiving intensive insulin therapy or continuous subcutaneous insulin infusion, had a screening HbA1c of ≤10%, a body mass index (BMI) of 19–30 kg/m^2^, C-peptide level of <0.8 ng/mL, and no communication barriers that would interfere with the education program. The exclusion criteria specified in the original study protocol encompassed gastroparesis, nephropathy, pregnancy, celiac disease, eating disorders, food allergy, gastrointestinal disease or malabsorption, and inability to adhere to study procedures. Continued CGM use was required for study follow-up. The absence of previous structured carbohydrate-counting education was established at screening by structured interview. Participants were asked whether they had ever completed a multi-session carbohydrate-counting program, whether they had ever been given an individualized ICR or ISF, and whether they routinely calculated prandial insulin doses from the carbohydrate content of meals. None of the participants had completed a multi-session carbohydrate-counting program or had been given an individualized ICR or ISF. Because prior education was established by self-report at a single screening interview, this criterion is stated here as the absence of a previous formal structured program rather than as the absence of any carbohydrate-related dose adjustment: as shown in Supplementary Table S4, 15 participants (50.0%) reported adjusting insulin when a meal contained more carbohydrate, 13 (43.3%) when a portion was larger and 11 (36.7%) for snack carbohydrate, so some participants were already making ad hoc prandial adjustments without a calculated ratio. Participants whose previous dietitian contact had consisted only of receiving a general meal plan were considered not to have received structured education; this accounts for the high proportion reporting previous dietitian contact in Table 1. Use of continuous subcutaneous insulin infusion was not taken as evidence of previous structured education, because pump therapy had been initiated at other centers without a structured carbohydrate-counting program.

Sixty individuals were assessed for eligibility, thirty-eight of whom entered this study. Of the 22 individuals who were not enrolled, most did not meet the eligibility criteria, and a few initially agreed to participate but subsequently declined; the number in each category was not recorded. Eight participants did not complete the follow-up, including six who discontinued CGM use and two who withdrew from further participation, leaving 30 participants in the primary analysis (Figure 1). Baseline data for the eight participants who did not complete follow-up were not retained in the study database, so completers and non-completers could not be compared; the analyses reported here are therefore completers analyses of 30 of the 38 enrolled participants. Because the study files did not record which clinic patients the eight non-completers were, their clinic records could not be identified, so the characteristics and reasons for discontinuation suggested by the reviewer could not be retrieved beyond those reported above (six discontinued CGM use and two withdrew). Eight participants whose baseline BMI was outside the prespecified eligibility range of 19–30 kg/m^2^ were enrolled in error; this protocol deviation was identified after the statistical analysis for the doctoral thesis had been completed. Their baseline BMI values ranged from 17.5 to 32.1 kg/m^2^, with four below and four above the eligibility range. Because these participants had completed the full intervention and follow-up, and because removing them after the analysis had been performed would itself have been a post hoc change to the analyzed sample, they were retained in the primary analysis. Because this protocol deviation affected more than a quarter of the analyzed sample, the sensitivity analysis restricted to the 22 participants who met the BMI criterion is reported alongside the primary analysis for every principal glycemic outcome (Supplementary Table S2). In that analysis the changes in TIR, TAR >250 mg/dL, mean sensor glucose, GMI, and HbA1c were in the same direction as in the primary analysis and reached nominal statistical significance, whereas for TAR 181–250 mg/dL the result was marginal (*p* = 0.040; 95% CI −8.86 to −0.23) and the change in CV was not statistically significant (*p* = 0.179); the change in TBR 54–69 mg/dL reached nominal significance in this subgroup (*p* = 0.026), which it did not in the primary analysis. A second eligibility deviation was also identified during review. One participant whose screening HbA1c met the ≤10% criterion had a baseline laboratory HbA1c of 12.3% measured at the first education visit; both the baseline value (12.3%) and the week-12 value (7.0%) were re-verified against the laboratory records for this revision and are correct as printed. The date of this participant’s screening HbA1c value was not recorded, so the interval between the screening and baseline measurements cannot be reported. This participant was likewise retained in the primary analysis, and a sensitivity analysis excluding them is reported for every principal glycemic outcome in Supplementary Table S5.

### 2.3. Sample Size

The ethics-approved protocol did not specify a fixed sample size; it anticipated 20–30 participants, consistent with comparable studies, with a formal power calculation to be performed after a pilot phase; no pilot phase was conducted. Recruitment was ultimately determined by the number of eligible adults with type 1 diabetes who attended the study center during the enrollment window and agreed to participate. A power calculation reported in the doctoral thesis, which indicated a minimum of 11 participants, was derived from a cross-sectional correlation rather than from an expected within-participant change in any outcome evaluated here and is therefore not used in this report. The study was therefore not powered for any specific outcome, and the 30 participants who completed follow-up constitute a convenience sample. Accordingly, precision is reported as 95% confidence intervals around the paired mean differences (Table 2) rather than as post hoc power.

### 2.4. Carbohydrate-Counting Education

One dietitian that specialized in diabetes delivered the education face-to-face, following the algorithms of the 2019 evidence-based nutrition therapy guideline of the Turkish Diabetes Dietitians Association [6]. The structured education and follow-up program comprised eight visits over 12 weeks, with more frequent visits during the initial education phase and progressively longer intervals during follow-up (Figure 2).

The structured three-level CCE program encompassed eight visits over 12 weeks. Baseline assessment was followed by progressive education covering carbohydrate-containing foods; portion estimation; personalized carbohydrate distribution; food-label reading; glycemic index; prandial glucose patterns; effects of protein, fat, and exercise; and tailored use and review of the ICR and ISF. Final assessments were completed at Visit 8.

At the first visit, Level 1 education encompassed macronutrients, the carbohydrate–insulin association, identification of carbohydrate-containing foods, the glycemic index concept, calculation of the carbohydrate content of foods, and portion estimation using a kitchen scale. At the second visit, the 3-day food record was reviewed, and a personalized daily carbohydrate target and its distribution across meals were agreed upon. The treating physician was consulted regarding insulin dose adjustment, and participants were instructed to consistently consume the agreed carbohydrate amounts, emphasizing low-glycemic-index carbohydrate sources, to facilitate insulin-dose evaluation and adjustment.

At the third visit, Level 2 education comprised food-label reading, interpretation of prandial glucose patterns in relation to the amount and type of carbohydrate consumed, the contribution of protein and fat to postprandial glycemia, and adjustment of carbohydrate intake or insulin dose according to physical activity type and intensity. Errors in carbohydrate estimation and discrepancies between meals were identified and corrected; topics were repeated when necessary. Participants progressed to Level 3 only after demonstrating satisfactory understanding of Level 2. At the fourth visit, carbohydrate intake and glucose patterns were reviewed, and insulin-dose adjustment was continued as needed.

Level 3 education was initiated at the fifth visit. Individualized ICRs, defined as the grams of carbohydrate covered by one unit of prandial insulin, and insulin sensitivity factors (ISFs), defined as the anticipated blood glucose reduction following one unit of insulin, were determined for each main meal; their application was practiced using worked examples. Meal-specific ICRs were derived clinically rather than by formula. Participants were asked to consume a consistent, individually agreed carbohydrate amount at each meal, and the treating physician reviewed the prandial insulin dose and the corresponding glucose values for each meal. When the increase from the preprandial value to the value 2 h after the meal exceeded 60 mg/dL, the bolus dose for that meal was increased by 1 unit; when 2 h postprandial glucose remained below 100 mg/dL, it was decreased by 1 unit. Each adjustment was decided by the physician after 3–4 days of monitoring. Once preprandial glucose had remained within 80–130 mg/dL and 2 h postprandial glucose at or below 180 mg/dL, with a preprandial-to-postprandial increase in no more than 60 mg/dL, for 1–2 weeks, the ICR for that meal was calculated by dividing the carbohydrate content of the meal (g) by the bolus dose (U). The ISF was calculated using the 1800 rule, by dividing 1800 by the total daily insulin dose at which glycemic regulation had been achieved. Subsequently, participants independently applied their individualized ratios for 15 days. At the sixth visit, the adequacy of the ICRs and ISFs was reviewed and revised when necessary, using the same criteria. At the seventh visit, participants’ use of the individualized ratios and carbohydrate-counting approach adherence were reviewed; further adjustments were made when required. At the eighth and final visit, at the end of the 12-week follow-up, anthropometric measurements, laboratory HbA1c, insulin-dose records, food records, and CGM data were reassessed.

Participants, including those with overweight or obesity, received no energy restriction or weight loss-related recommendation as part of the intervention. They were instructed to maintain their habitual physical activity throughout the study. Because the program was delivered alongside routine clinical care, insulin therapy was adjusted by the treating physician during follow-up; these adjustments are a co-intervention and cannot be separated from the education itself. All 16 participants using continuous subcutaneous insulin infusion were on non-automated pump therapy; none used an automated insulin delivery (closed-loop) system. Individual dose adjustments made by the treating physician during follow-up were not systematically recorded; group-level basal, bolus, and total daily insulin doses at baseline and week 12 are reported in Table 3. Severe hypoglycemia and diabetic ketoacidosis were ascertained at each study visit; no dedicated prospective adverse-event reporting protocol was used. The unchanged time below range therefore does not constitute a complete assessment of intervention safety.

### 2.5. CGM Metrics

Participants used FreeStyle Libre 1 (Abbott Diabetes Care, Alameda, CA, USA) or Dexcom G6 (Dexcom, Inc., San Diego, CA, USA) continuous glucose monitoring (CGM) systems. Fourteen participants (46.7%) used FreeStyle Libre 1 and 16 (53.3%) used Dexcom G6, and each participant used the same system at both assessments. The device counts were taken from the sensor download records; although they coincide numerically with the numbers of participants using MDI and CSII, the two variables do not coincide at the participant level: of the Dexcom G6 users, 12 were on CSII and 4 on MDI, and of the FreeStyle Libre 1 users, 4 were on CSII and 10 on MDI (Supplementary Table S8). Because the two systems differ in measurement accuracy and in alarm functionality, which may affect the TBR and TIR estimates in particular, the principal glycemic outcomes were also analyzed separately by system (Supplementary Table S8). Fourteen-day CGM reports covering the period immediately before the first education visit and the period immediately before the final visit were downloaded from the web-based platform of each participant’s sensor system. In accordance with the international consensus [13], only reports with at least 70% sensor-active time over the 14-day period were analyzed; mean sensor-active time was 92.3% ± 8.3% (range 77–100%) at baseline and 91.4% ± 8.7% (range 70–100%) at week 12. The following metrics were extracted: TIR (70–180 mg/dL), TAR (181–250 and >250 mg/dL), TBR (54–69 and <54 mg/dL), CV as a glycemic variability measure, mean sensor glucose, and glucose management indicator (GMI). CGM metrics were interpreted according to the abovementioned consensus targets [13,14].

### 2.6. Anthropometric and Insulin Data

Body weight was measured using a bioelectrical impedance analyzer (TBF-300; Tanita Corporation, Tokyo, Japan), and BMI was calculated from the measured weight and height. HbA1c was measured by high-performance liquid chromatography (HPLC) using the D-10™ Hemoglobin Testing System (Bio-Rad Laboratories, Inc., Hercules, CA, USA). Laboratory-measured HbA1c and daily basal, bolus, and total insulin doses were obtained from the center’s patient follow-up records at baseline and at the end of the 12-week follow-up. ICRs, individually identified during Level 3 of the carbohydrate-counting education, were separately recorded for breakfast, lunch, and dinner, together with each participant’s ISF. For exploratory comparison, ICRs were also estimated using the conventional 500 and 450 rules based on total daily insulin dose.

### 2.7. Dietary Assessment and Baseline Questionnaire

Participants completed a 3-day food record, including two weekdays and one weekend day, at baseline and week 12. Energy and nutrient intakes were calculated using Ebispro for Windows (Turkish Version BeBiS 8.2; Pasifik Elektrik Elektronik Ltd. Şti., Istanbul, Türkiye; 2019). Standard Turkish recipe references were employed for estimating the nutrient composition of mixed dishes when necessary [19,20]. Dietary intakes were evaluated with reference to the Turkey Dietary Guidelines 2022 [21].

At baseline, a 22-item questionnaire was administered to collect demographic data and assess diabetes-related dietary and self-management practices, including hypoglycemia management, meal patterns, physical activity, and use of CGM data.

### 2.8. Statistical Analysis

Categorical variables were presented as frequencies and percentages, and continuous variables as means ± standard deviations; variables compared with the Wilcoxon signed-rank test are additionally presented as medians with interquartile ranges in Supplementary Table S3. The Shapiro–Wilk test was used to assess the distribution of each variable at baseline and at week 12, and the paired-samples *t*-test was applied when both distributions were compatible with normality and the Wilcoxon signed-rank test otherwise; the test used for each variable is identified in the footnote to Table 2 and in Table 3. TIR (70–180 mg/dL) was designated as the primary outcome; this designation was made after data collection and analysis and is therefore post hoc. TAR, TBR, CV, mean sensor glucose, GMI, and HbA1c were secondary glycemic outcomes, and body weight, insulin dose, dietary intake, and the ICR comparison were exploratory. Because more than ten outcomes were examined, no formal adjustment for multiple comparisons was applied; all p-values other than that for the primary outcome are nominal and should be interpreted as hypothesis-generating. Paired mean differences are reported with 95% confidence intervals to convey precision. Baseline dependence of the change in TIR was examined by correlating the change with the baseline value and, because this correlation is biased toward negative values by mathematical coupling, also with the mean of the baseline and week-12 values (Oldham’s method) [22], and by regressing week-12 TIR on baseline TIR. Oldham’s correlation is, however, algebraically equivalent to a test of whether the variance of TIR changed between the two assessments, and regression to the mean produces exactly that change in variance; neither this correlation nor a baseline-adjusted regression can therefore separate a genuine effect from regression to the mean in a single-arm design, and both are reported as descriptions of the baseline dependence of the change [23]. For every outcome compared with the Wilcoxon signed-rank test, the Hodges–Lehmann estimate of the median paired difference is reported with its distribution-free 95% confidence interval [24], so that the point estimate, the interval and the *p*-value all derive from the same test (Supplementary Table S3). Because selecting the test from the Shapiro–Wilk result is itself data-dependent at this sample size, the paired-samples *t*-test was additionally applied to every outcome as a sensitivity check, and both *p*-values are reported for all outcomes in Supplementary Table S6. Energy-intake plausibility was assessed by expressing reported energy intake as a multiple of basal metabolic rate estimated from the Schofield weight-based equations [25], and comparing it with the Goldberg cut-off of 1.35 [26]. A sensitivity analysis restricted to participants whose baseline BMI was within the prespecified eligibility range of 19–30 kg/m^2^ was performed for the principal glycemic outcomes. All tests were two-sided, with *p* < 0.05 considered statistically significant. Analyses were performed using Statistical Package for the Social Sciences (version 27; IBM Corp., Armonk, NY, USA). For this revision, every value reported in Table 1, Table 2 and Table 3 and in Supplementary Tables S1–S7 was recomputed from the de-identified participant-level dataset, which is provided together with the analysis syntax as supplementary files. The statistician named in the Acknowledgments re-ran these analyses with the syntax in SPSS version 27 and confirmed the values reported in Table 1, Table 2 and Table 3 and Supplementary Tables S1–S8.

## 3. Results

### 3.1. Participant Characteristics and Study Flow

Of the 38 participants who entered this study, 30 completed the 12-week follow-up and were included in the primary analysis. The final sample comprised 23 females and 7 males (mean age, 35.9 ± 10.6 years; mean diabetes duration, 12.4 ± 8.5 years). All participants had a university education, 46.7% received multiple daily injections, 53.3% had continuous subcutaneous insulin infusion, and 90.0% had previously consulted a dietitian (Table 1). Of the 27 participants with previous dietitian contact, 17 had attended a single consultation and 10 had attended more than one; in all cases this contact had consisted of receiving a general meal plan, and none had completed a structured carbohydrate-counting program. Findings from the baseline questionnaire are presented in Supplementary Table S4.

### 3.2. Changes in Glycemic Outcomes

TIR (70–180 mg/dL), the primary outcome, increased from 62.53% ± 14.93% at baseline to 72.97% ± 10.88% at week 12 (mean difference +10.43 percentage points, 95% CI +7.07 to +13.80; *p* < 0.001). TAR decreased from 24.67% ± 11.01% to 19.37% ± 8.25% for the 181–250 mg/dL range (*p* = 0.002) and from 8.90% ± 7.63% to 4.63% ± 4.19% for >250 mg/dL values (*p* < 0.001). Furthermore, the mean sensor glucose decreased from 159.30 ± 26.10 to 147.87 ± 17.05 mg/dL (*p* < 0.001) (Table 2; Figure 3). Paired mean differences with 95% confidence intervals for all glycemic outcomes are presented in Table 2. Because TIR was compared with the Wilcoxon signed-rank test, the corresponding Hodges–Lehmann median difference and its distribution-free interval are also reported: +10.00 percentage points (95% CI +7.00 to +12.50). A bootstrap confidence interval for the mean difference (+7.4 to +13.8 percentage points) was almost identical to the t-based interval, and Hodges–Lehmann estimates for every outcome tested with the Wilcoxon signed-rank test are given in Supplementary Table S3. The increase in TIR was inversely related to baseline TIR (Pearson r = −0.690, *p* < 0.001; Spearman ρ = −0.782, *p* < 0.001), and the standard deviation of TIR fell from 14.93 to 10.88 percentage points. The inverse relationship persisted when the change was related to the mean of the baseline and week-12 values (Oldham’s method: Pearson *r* = −0.473, *p* = 0.008). This correlation is, however, algebraically equivalent to a test of whether the variance of TIR changed between the two assessments, and the variance did fall; because regression to the mean produces exactly this pattern, Oldham’s method does not establish that the baseline dependence is more than mathematical coupling. In a baseline-adjusted model, week-12 TIR regressed on baseline TIR gave a slope of 0.58 (95% CI 0.42 to 0.75), significantly below unity (*p* < 0.001), which is likewise what regression to the mean predicts; because the fitted line passes through the sample means, this model reproduces the mean change of +10.43 percentage points and cannot separate a genuine effect from regression to the mean in the absence of a parallel control group. Descriptively, mean TIR increased by 14.4 percentage points in the 19 participants whose baseline TIR was below 70% and by 3.6 percentage points in the 11 who were already at or above the target (95% CI −0.2 to +7.5; *p* = 0.063); this subgroup split was not prespecified, the smaller change in the at-target group was not statistically significant, and both figures are reported as description only.

The CV was lower at week 12 than at baseline (35.66% ± 4.54% vs. 33.56% ± 4.49%; *p* = 0.029). Laboratory-measured HbA1c decreased from 7.71% ± 1.17% to 7.08% ± 0.57% (mean difference −0.63 percentage points, 95% CI −1.03 to −0.23; *p* < 0.001), whereas the sensor-derived GMI decreased from 7.10% ± 0.63% to 6.85% ± 0.40% (*p* = 0.001; Table 2). The fall in laboratory HbA1c (−0.63 percentage points) therefore exceeded the fall in GMI (−0.26 percentage points), and baseline laboratory HbA1c was approximately 0.6 percentage points higher than baseline GMI. GMI was taken from each participant’s sensor report rather than calculated from the tabulated mean sensor glucose. Device-reported GMI is given to one decimal place and is derived from the sensor system’s own mean glucose, so it is not an exact linear transformation of the mean glucose values in Table 2: the mean sensor glucose change of −11.43 mg/dL implies a GMI change of −0.27 percentage points under the published GMI equation, against the −0.26 percentage points observed, and the two changes are closely correlated (*r* = 0.937). This rounding, together with differences of up to 0.47 percentage points in five participants between the device-reported GMI and the GMI implied by the tabulated mean sensor glucose, also accounts for the difference between the two *p*-values (*p* < 0.001 for mean sensor glucose and *p* = 0.001 for GMI in the primary analysis; *p* = 0.007 and *p* = 0.009 respectively in the sensitivity analysis). Both variables were recomputed from the sensor reports for this revision and no transcription error was found. One participant had a baseline laboratory HbA1c of 12.3% despite a screening value within the eligibility limit of ≤10%; when this participant was excluded, HbA1c decreased by 0.47 percentage points (95% CI −0.70 to −0.24; *p* < 0.001), and its standard deviation narrowed from 0.80 to 0.58. This participant’s screening HbA1c met the ≤10% eligibility criterion, whereas the baseline laboratory value measured at the first education visit was 12.3%; both values were re-verified against the laboratory records, and this is reported as a second eligibility deviation in Section 2.2. A sensitivity analysis excluding this participant is presented for every principal glycemic outcome in Supplementary Table S5: TIR increased by 10.28 percentage points (95% CI +6.81 to +13.75), GMI fell by 0.23 percentage points (95% CI −0.37 to −0.09), and the direction and statistical significance of every other glycemic outcome were unchanged. In the sensitivity analysis by CGM system (Supplementary Table S8), TIR increased by a similar amount with Dexcom G6 (+10.56 percentage points, 95% CI +5.33 to +15.79; *n* = 16) and with FreeStyle Libre 1 (+10.29 percentage points, 95% CI +5.46 to +15.11; *n* = 14), and neither TBR category changed significantly with either system; by contrast, the lower CV at week 12 was confined to FreeStyle Libre 1 users (−4.26 percentage points, 95% CI −6.78 to −1.75), with no difference among Dexcom G6 users (−0.19 percentage points, 95% CI −2.79 to +2.40). TBR demonstrated no significant changes: TBR 54–69 mg/dL changed from 3.53% ± 3.72% to 2.67% ± 2.34% (*p* = 0.133), and TBR <54 mg/dL from 0.47% ± 0.73% to 0.40% ± 0.50% (*p* = 0.564). No episodes of severe hypoglycemia or diabetic ketoacidosis occurred during the 12-week follow-up.

The sensitivity analysis restricted to participants who met the prespecified baseline BMI eligibility criterion of 19–30 kg/m^2^ (*n* = 22) showed changes in the same direction as the primary analysis for every principal glycemic outcome. The changes in TIR, TAR >250 mg/dL, mean sensor glucose, GMI, and laboratory-measured HbA1c reached nominal statistical significance in this subgroup, whereas the change in TAR 181–250 mg/dL was marginal (−4.55 percentage points, 95% CI −8.86 to −0.23; *p* = 0.040) and the change in CV was not statistically significant (−1.54 percentage points, 95% CI −3.83 to +0.76; *p* = 0.179). The change in TBR 54–69 mg/dL, which was not statistically significant in the primary analysis, reached nominal significance in this subgroup (−1.41 percentage points; *p* = 0.026). Because this subgroup comprises 22 of the 30 analyzed participants, these analyses are not independent of the primary analysis and are reported to show how sensitive each estimate is to the protocol deviation rather than as confirmation of it (Supplementary Table S2).

### 3.3. Changes in Body Weight, Insulin Dose, and Dietary Intake

Body weight did not change significantly from baseline to week 12 (68.73 ± 14.93 vs. 68.89 ± 14.33 kg; *p* = 0.741), nor did BMI (24.22 ± 4.41 vs. 24.25 ± 4.04 kg/m^2^; *p* = 0.854). Total daily insulin dose did not change significantly (41.53 ± 13.15 vs. 39.53 ± 14.15 U/day; *p* = 0.115), nor did basal (20.83 ± 8.10 vs. 20.40 ± 8.22 U/day; *p* = 0.289) or bolus insulin dose (20.70 ± 6.56 vs. 19.13 ± 7.89 U/day; *p* = 0.102) (Table 3). These nonsignificant results indicate that an increase in insulin requirement was not detected; with 30 participants they do not establish that insulin dose was unchanged.

Over the 12-week follow-up, energy and macronutrient intakes did not change significantly. Energy intakes were 1629.38 ± 414.56 and 1591.37 ± 378.20 kcal/day at baseline and week 12, respectively (*p* = 0.432). No significant changes were noted in carbohydrate intake (156.00 ± 54.57 vs. 153.32 ± 51.21 g/day; *p* = 0.598), carbohydrate as a percentage of energy (39.07% ± 6.29% vs. 39.61% ± 7.21%; *p* = 0.647), protein intake (71.09 ± 17.37 vs. 71.11 ± 18.40 g/day; *p* = 0.994), fat intake (77.78 ± 20.12 vs. 74.58 ± 19.08 g/day; *p* = 0.383), or fiber intake (22.84 ± 5.44 vs. 23.93 ± 6.97 g/day; *p* = 0.324) (Table 3). Reported energy intake corresponded to 1.09 ± 0.20 times the estimated basal metabolic rate at baseline and 1.06 ± 0.21 at week 12. Twenty-six of the 30 participants (87%) at baseline and 27 (90%) at week 12 fell below the Goldberg cut-off of 1.35, indicating that under-reporting was widespread rather than confined to a few records [26]. Meal-level carbohydrate distribution and the glycemic index of the carbohydrate consumed were educational targets of the program but were not quantified in the food records and are therefore not reported. Findings from the 22-item baseline questionnaire are presented in Supplementary Table S4. Because reported energy intake was implausibly low for almost every participant, the comparison of the reported macronutrient distribution with the Turkey Dietary Guidelines 2022 is presented in Supplementary Table S7 and is not interpreted here.

### 3.4. Individualized ICRs

At the end of the structured education program, the individualized mean ICR was 7.90 ± 1.66 g/U. Meal-specific ICRs at breakfast, lunch, and dinner were 6.47 ± 1.22, 8.53 ± 2.13, and 8.70 ± 2.15 g/U, respectively. The mean ISF was 49.17 ± 15.03 mg/dL/U. Expressed as medians, the ICRs were 6.0 g/U (interquartile range 5.8–8.0) at breakfast, 8.0 g/U (7.0–10.0) at lunch, and 8.0 g/U (7.0–10.0) at dinner, corresponding to median doses of approximately 1.7, 1.3, and 1.3 units of prandial insulin per 10 g of carbohydrate (Supplementary Table S1).

For comparison, conventional formula-based ICR estimates calculated using postintervention total daily insulin dose were 13.95 ± 4.01 and 12.56 ± 3.61 g/U using the 500 and 450 rules, respectively (Supplementary Table S1). Both formula-based estimates exceeded the individually determined mean daily ratio (mean difference +6.05 g/U for the 500 rule and +4.66 g/U for the 450 rule; Wilcoxon *p* < 0.001 for both), corresponding to ratios approximately 1.8 and 1.6 times the measured values. The limits of agreement were wide (−1.53 to +13.63 g/U for the 500 rule and −2.21 to +11.53 g/U for the 450 rule), so agreement between the two quantities was poor at the individual level (Supplementary Table S1). Because the 500 and 450 rules are intended only as starting estimates for subsequent titration, whereas the individually determined ratio is derived from the bolus dose that titration eventually reached, this comparison describes the distance between a starting estimate and the titrated result rather than the accuracy of either quantity. Because both formula-based estimates are derived from the postintervention total daily dose, they are not independent of the insulin doses in use at the end of the program. The individually determined ratios were established clinically and were not separately validated against postprandial glucose outcomes, so neither approach can serve as a reference standard here. The difference between the two approaches was more pronounced among female participants, and the small number of male participants (*n* = 7) precludes any sex-specific inference (Supplementary Table S1).

## 4. Discussion

An association was noted between structured carbohydrate-counting education and improved glycemic outcomes over 12 weeks in adults with type 1 diabetes who were already using CGM. TIR, the primary outcome, increased by 10.4 percentage points, whereas both TAR categories and laboratory-measured HbA1c decreased and CV was lower at week 12, with TBR, total daily insulin dose, and body weight exhibiting no significant increases. Furthermore, energy and macronutrient intakes remained largely unchanged. Because the study was uncontrolled, these findings describe the change observed over the program rather than an effect attributable to it; they are consistent with the possibility that structured carbohydrate-counting education supports glycemic management beyond HbA1c alone, particularly through TIR, and this requires confirmation in a controlled design.

Reductions in both TAR categories and a 0.63-percentage-point reduction in laboratory-measured HbA1c accompanied the 10.4-percentage-point increase in TIR. Earlier studies and reviews have indicated variable effects of carbohydrate-counting interventions on glycemic control in adults with type 1 diabetes [8,27,28,29,30,31]. More recent evidence has extended these findings to CGM-derived outcomes. A 2025 systematic review and meta-analysis of telemedicine-delivered carbohydrate-counting interventions revealed a mean 9.59-percentage-point increase in TIR alongside a modestly reduced HbA1c [15]. Likewise, structured nutrition education accompanying glucose-sensor initiation was associated with higher TIR and lower HbA1c at 12 months in adults with type 1 diabetes [16], whereas a recent randomized trial of an application-based carbohydrate-counting and bolus-calculation intervention also reported promising effects on TIR [17]. Although variations in study design, intervention delivery, and concurrent technology use preclude direct comparison, the direction of these findings aligns with the TIR improvement observed in the present study.

Moreover, the observed changes align with established TIR–HbA1c associations [32,33]. At the group level, mean TIR increased from below to above the recommended 70% target, whereas mean TAR >250 mg/dL decreased to below the recommended 5% threshold. These targets remain incorporated into current clinical standards for CGM assessment [13,14]. Notably, these improvements occurred without a substantially elevated TBR. Collectively, these findings indicate a shift toward a more favorable overall CGM profile rather than improvement in average glycemia alone.

Furthermore, the CV was lower at week 12 than at baseline (35.66% to 33.56%), although mean baseline CV was already below the recommended ≤36% threshold, so the group did not move from above to below the target. CV offers information on glucose dispersion that complements TIR and HbA1c; current CGM standards recommend a CV of ≤36% for most individuals with diabetes [13,14]. Evidence regarding the effects of carbohydrate-counting interventions, particularly on glycemic variability, remains scarce. A randomized trial reported that an intervention combining automated bolus calculation with flash glucose monitoring decreased CV in adults with type 1 diabetes [34]. In the present study the lower CV at week 12 reached nominal statistical significance in the primary analysis but not in the sensitivity analysis restricted to participants who met the prespecified BMI eligibility criterion, in which the change was in the same direction but was not statistically significant; it was also confined to users of one CGM system (FreeStyle Libre 1; Supplementary Table S8). Therefore, the finding regarding glycemic variability requires cautious interpretation and confirmation in larger controlled studies.

The improvements in glycemic outcomes occurred without a detected increase in total daily insulin dose or body weight. Previous studies and reviews have suggested that increases in insulin requirements or body weight do not necessarily accompany improvements in glycemic control following carbohydrate-counting interventions [28]. During this study, no energy restriction or weight-loss intervention was prescribed. The absence of a detected increase in insulin requirement or body weight alongside improved glycemic outcomes may be clinically relevant when structured carbohydrate counting is considered as part of medical nutrition therapy; with 30 participants, however, these nonsignificant comparisons do not demonstrate that insulin dose and body weight were truly unchanged. A further consideration is that the observed fall in laboratory HbA1c (−0.63 percentage points) was larger than the fall in GMI (−0.26 percentage points), and baseline laboratory HbA1c exceeded baseline GMI by approximately 0.6 percentage points. GMI is calculated from mean sensor glucose, and estimates, rather than measures, glycation; discrepancies of this size between measured HbA1c and GMI are well recognized and reflect between-individual differences in red-cell turnover and glycation rate, as well as the fact that the 14-day CGM window and the preceding 8–12 weeks of erythrocyte exposure captured by HbA1c cover different periods [35]. Part of the difference in the present study was attributable to a single participant whose laboratory HbA1c fell from 12.3% to 7.0%; without this participant, laboratory HbA1c decreased by 0.47 percentage points, closer to the change in GMI. The larger decline in laboratory HbA1c than in GMI should therefore be interpreted with caution and does not necessarily indicate a greater improvement than that captured by CGM.

The discrepancy between individually determined ICRs and conventional formula-derived estimates represents an exploratory finding of this study. The mean individualized ICR was 7.90 g/U, compared with 13.95 and 12.56 g/U using the 500 and 450 rules, respectively, with meal-specific individualized ratios being the lowest at breakfast. Similarly, previous studies have demonstrated differences between formula-derived and individually determined ICRs, as well as meal-specific variation in insulin requirements [9,10]. This comparison is, however, close to a restatement of the titrated insulin dose. The individually determined ratio is a fixed agreed carbohydrate amount divided by the clinician-titrated bolus dose, and dividing the mean reported carbohydrate intake at week 12 by the mean individualized ratio (153.32 g ÷ 7.90 g/U) returns 19.41 U/day, almost exactly the observed mean week-12 bolus dose of 19.13 U/day. The 500 and 450 rules, by contrast, are derived from the total daily dose and were only ever intended as starting estimates to be titrated. The difference between the two therefore describes the distance between a starting estimate and the dose eventually reached rather than the accuracy of either; this study was not designed to validate an ICR formula, and these findings are reported descriptively, with no conclusion drawn about either approach.

Although glycemic outcomes improved, no significant changes in reported energy or macronutrient intake were observed over the program. Carbohydrate counting mainly offers a framework for managing carbohydrate intake with prandial insulin dosing and should not be assumed to address all dietary quality dimensions. Previous studies in individuals with type 1 diabetes have revealed suboptimal adherence to healthy-eating recommendations and dietary patterns that may not fully align with nutritional guidance [36,37,38,39]. These findings underscore the significance of incorporating carbohydrate-counting education into a comprehensive medical nutrition therapy that also addresses overall dietary quality and personalized nutrition needs. In the present study the reported macronutrient distribution differed from the ranges recommended by the Turkey Dietary Guidelines 2022 at both time points (Supplementary Table S7), but because reported energy intake was implausibly low for almost every participant these records cannot support any inference about habitual macronutrient distribution or about whether the program changed it. This dietary interpretation is nevertheless constrained by the method of assessment. Reported energy intake averaged 1.09 times the estimated basal metabolic rate, and 87% of participants fell below the Goldberg cut-off of 1.35 at baseline (1.06 and 90%, respectively, at week 12), indicating substantial and near-universal under-reporting of the food records [26]. Because under-reporting of this magnitude may also differ between baseline and follow-up, the absence of a detected change in intake should not be read as evidence that eating behavior was unaltered, particularly because the meal-level distribution of carbohydrate and the glycemic index of the carbohydrate consumed, both explicit targets of the program, were not quantified.

Several strengths and limitations should be considered when interpreting these findings. All participants were using CGM before enrollment, enabling glycemic outcome evaluation without the concurrent introduction of sensor technology, and a single diabetes dietitian delivered the structured education program using a standardized approach.

However, the single-arm pre–post design without a parallel control group precluded attributing the observed changes specifically to the education program, as regression to the mean, increased clinical attention, or other time-related factors may have also contributed. A concurrent control group was not included because, as stated in the ethics-approved protocol, withholding carbohydrate-counting education from patients attending for diabetes care was considered ethically inappropriate; a wait-list or delayed-education control design would allow this limitation to be addressed in future studies. Regression to the mean is a particular concern here: the increase in TIR was strongly and inversely related to baseline TIR (r = −0.690), the standard deviation of TIR narrowed from 14.93 to 10.88 percentage points, and that of HbA1c from 1.17 to 0.57, while screening excluded anyone with an HbA1c above 10%; one participant whose baseline laboratory HbA1c (12.3%) exceeded the screening value accounted for much of the narrowing in HbA1c dispersion, although the reduction in HbA1c remained statistically significant without this participant; the full sensitivity analysis excluding this participant is reported in Supplementary Table S5. Neither Oldham’s method nor a baseline-adjusted regression of week-12 TIR on baseline TIR can separate regression to the mean from a genuine effect in a single-arm design, because both are determined by the same change in variance. Enrollment extended over approximately twelve months, and each participant’s baseline and week-12 CGM windows were 12 weeks apart and therefore often fell in different seasons; seasonal variation in physical activity and eating patterns may thus have contributed independently of the program. In addition, the eight-visit schedule itself represented considerably more frequent clinical contact than routine care, and this added contact cannot be separated from the educational content. Insulin therapy was additionally adjusted by the treating physician during follow-up, and the individualized ICR and ISF were revised at later visits; these co-interventions are inseparable from the education itself in this design, and because individual dose adjustments were not systematically recorded, their extent cannot be quantified. The proposed mechanism was also not tested directly: carbohydrate-counting accuracy was not measured before or after the program, and daytime, postprandial, and overnight TIR could not be analyzed separately because complete raw sensor data and meal annotations were not available, so it cannot be established that the glycemic improvement arose from better meal-time carbohydrate–insulin matching rather than from other aspects of intensified care. The study was also registered retrospectively and was not formally powered for any outcome, and eight of the 38 enrolled participants did not complete follow-up, so attrition may have selected for participants who were more engaged with CGM; because baseline data for these participants were not retained, this possibility could not be examined. The sample was small, predominantly female, and restricted to university-educated adults, potentially limiting generalizability, and the 12-week follow-up failed to establish whether the observed changes were sustained over time. Additionally, dietary intake was assessed using self-reported three-day food records, in which under-reporting was widespread, and CGM systems were not standardized across participants, although each participant used the same system at both assessments; the change in TIR was similar with both CGM systems, but the lower CV at week 12 was confined to FreeStyle Libre 1 users, and the subgroups by system were too small to exclude system-related differences, particularly in TBR and CV (Supplementary Table S8). A protocol deviation also occurred: eight participants were enrolled in error despite baseline BMI values outside the prespecified eligibility range. Although sensitivity analyses restricted to participants meeting the BMI criterion showed changes in the same direction for TIR, both TAR categories, mean sensor glucose, GMI, and HbA1c, the change in TAR 181–250 mg/dL was only marginally significant in that subgroup and the change in CV was not statistically significant. Therefore, the finding regarding glycemic variability warrants cautious interpretation. Finally, more than ten outcomes were compared without adjustment for multiplicity, so the secondary *p*-values should be regarded as hypothesis-generating.

To confirm the observed changes in CGM-derived outcomes and identify their durability, larger randomized controlled studies with parallel comparison groups and longer follow-up are warranted; such studies should also measure carbohydrate-counting accuracy directly and report postprandial and overnight glucose metrics separately, so that the mechanism of any benefit can be identified. Moreover, future studies that prospectively evaluate individualized meal-specific ICRs and determine whether their use offers additional glycemic benefit compared with conventional formula-based estimates are needed. Overall, structured dietitian-led carbohydrate-counting education was associated with improved TIR and other glycemic outcomes over 12 weeks in adults with type 1 diabetes already using CGM, without a detected increase in TBR, insulin requirements, or body weight. Controlled studies are required to determine the extent to which these changes can be attributed specifically to the education intervention.

## 5. Conclusions

In this uncontrolled single-arm study, structured dietitian-led carbohydrate-counting education was associated with improved TIR, lower TAR, and lower HbA1c over 12 weeks in adults with type 1 diabetes already using CGM, without a detected increase in TBR, total daily insulin dose, or body weight. The lower glycemic variability at week 12 reached nominal statistical significance in the primary analysis but not in the sensitivity analysis, and mean baseline variability was already below the recommended threshold, so this finding should be interpreted with caution. Reported energy and macronutrient intakes did not change significantly, although under-reporting limits this inference. Individually determined meal-specific ICRs differed substantially from 500- and 450-rule estimates; this comparison was exploratory and does not establish the superiority of either approach. Because the design included no control group and insulin therapy was concurrently adjusted, these changes cannot be attributed specifically to the education program, and the findings should be regarded as hypothesis-generating. They are consistent with, but do not by themselves establish, a role for structured carbohydrate-counting education within comprehensive medical nutrition therapy for adults with type 1 diabetes. Adequately powered controlled studies with longer follow-up are required.

## Figures and Tables

**Figure 1:**
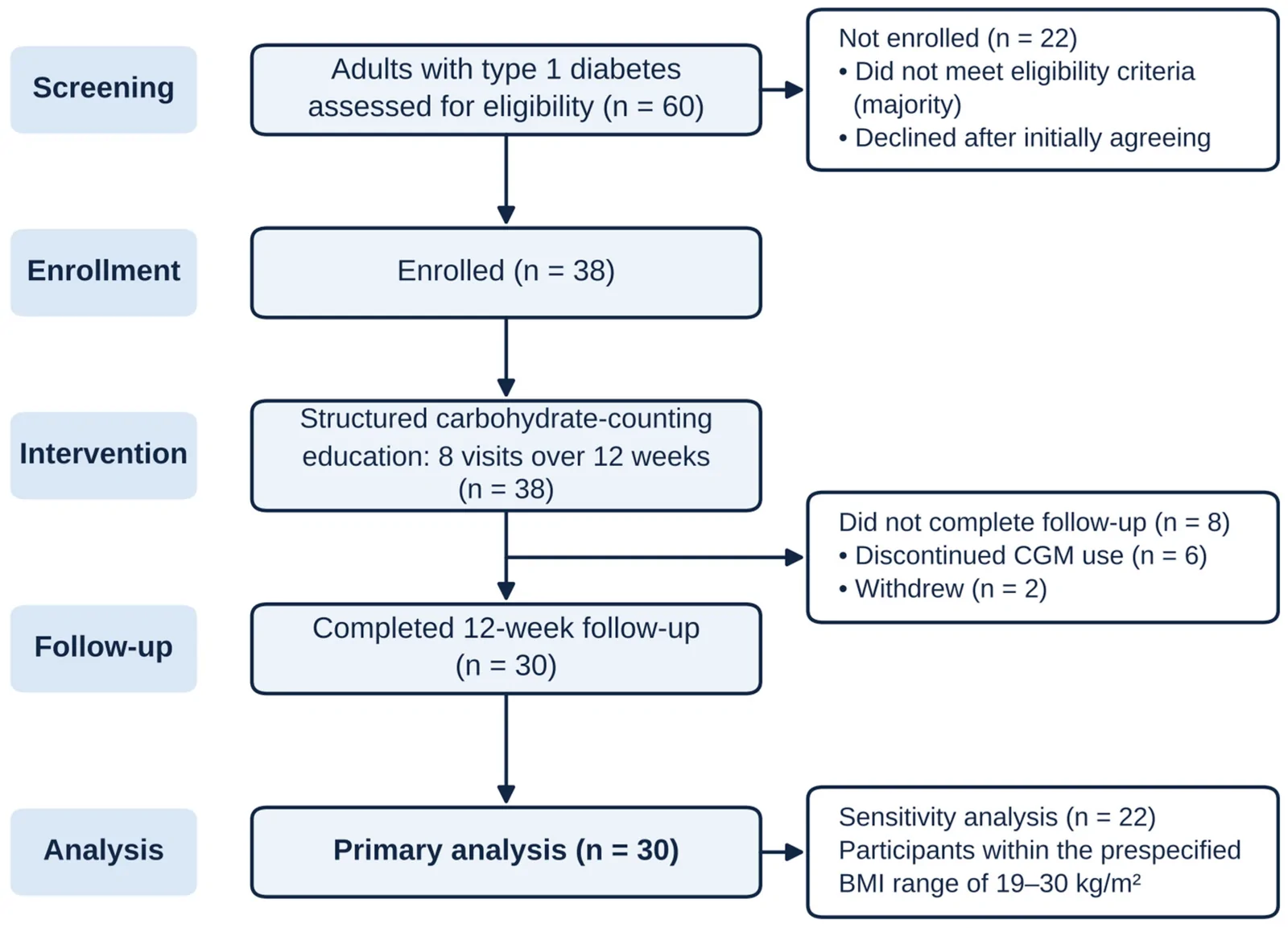
Participant flow through the study. Sixty individuals were assessed for eligibility, 38 were enrolled and 30 completed the 12-week follow-up; of the eight who did not complete follow-up, six discontinued CGM use and two withdrew. All analyses are completers analyses restricted to the 30 participants who completed follow-up.

**Figure 2:**
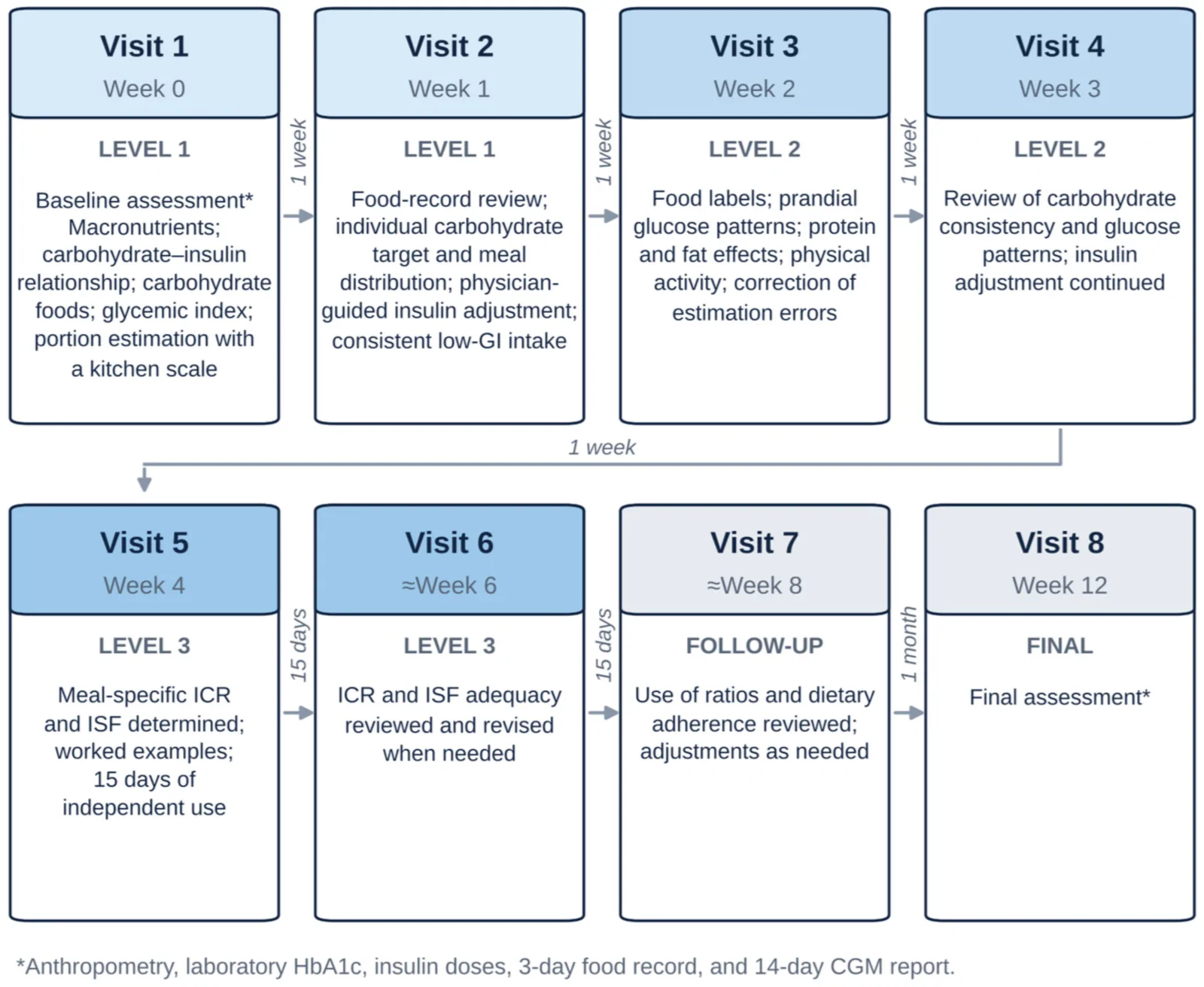
Structured individualized carbohydrate-counting education (CCE) process. CGM, continuous glucose monitoring; ICR, insulin-to-carbohydrate ratio; ISF, insulin sensitivity factor; low-GI, low glycemic index.

**Figure 3:**
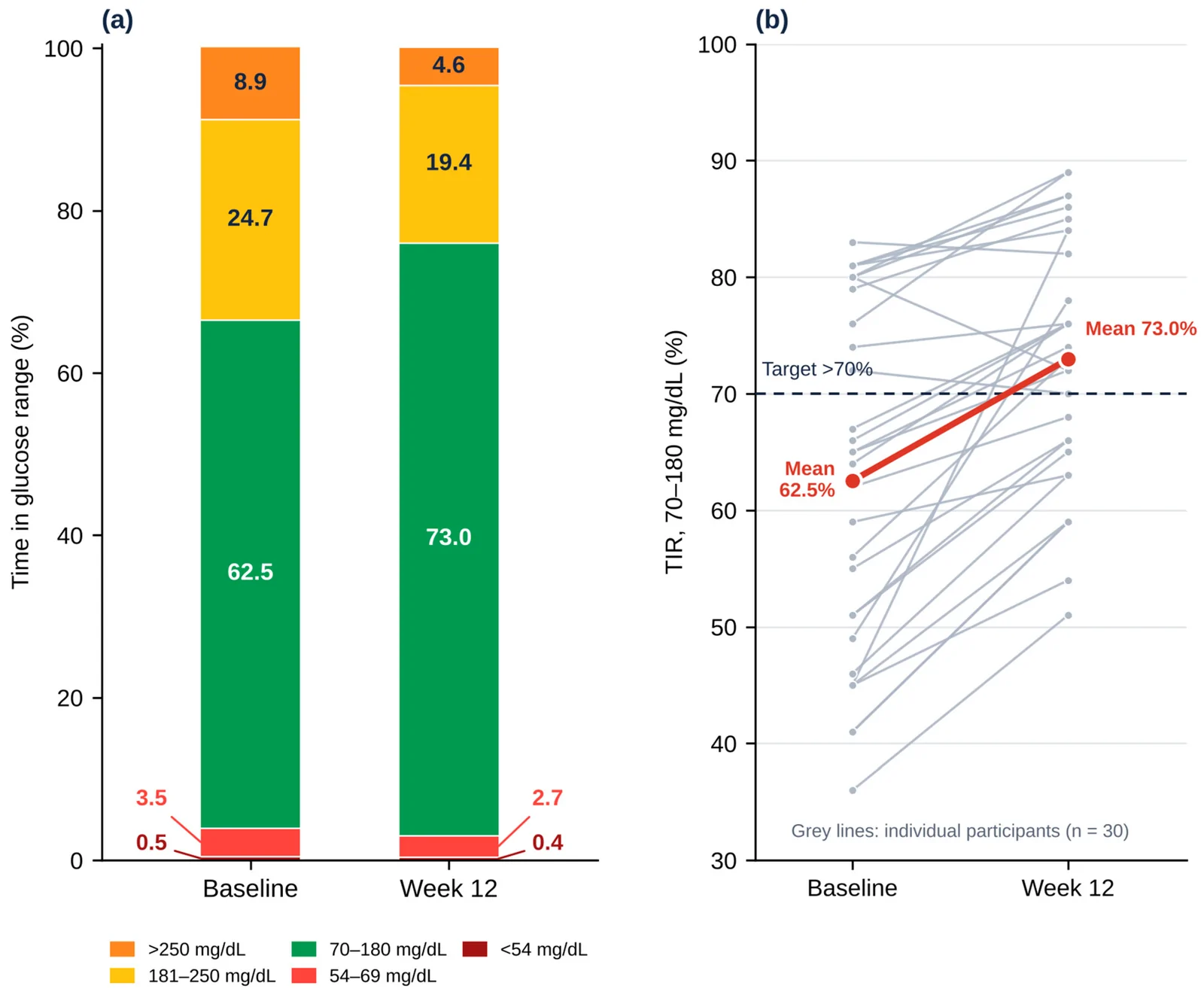
Changes in continuous glucose monitoring (CGM) metrics following the 12-week structured carbohydrate-counting education. (**a**) Mean percentage of time spent in each consensus glucose range at baseline and week 12; values for the two time-below-range components are labelled beside the bars. (**b**) Individual participant changes in TIR (70–180 mg/dL); the red line shows the group mean. TIR increased from 62.5% to 73.0% (*p* < 0.001). The dashed line denotes the consensus TIR target of >70%. CGM, continuous glucose monitoring; TBR, time below range; TIR, time in range.

**Table 1:** Baseline characteristics of the study participants (*n* = 30).

Characteristic	Value
Female, *n* (%)	23 (76.7)
Male, *n* (%)	7 (23.3)
Age, years	35.93 ± 10.55
Duration of diabetes, years	12.40 ± 8.51
Body weight, kg	68.73 ± 14.93
BMI, kg/m^2^	24.22 ± 4.41
HbA1c, %	7.71 ± 1.17
HbA1c, mmol/mol	61 ± 13
MDI therapy, *n* (%)	14 (46.7)
CSII therapy, *n* (%)	16 (53.3)
Total daily insulin dose, U/day	41.53 ± 13.15
TIR (70–180 mg/dL), %	62.53 ± 14.93
CV, %	35.66 ± 4.54
Previous dietitian consultation, *n* (%)	27 (90.0)

Data are presented as means ± SDs or *n* (%). HbA1c is reported in NGSP units (%) and IFCC units (mmol/mol); mmol/mol values are converted from HbA1c (%). BMI, body mass index; CSII, continuous subcutaneous insulin infusion; CV, coefficient of variation; MDI, multiple daily injections; TIR, time in range; SD, standard deviation.

**Table 2:** Changes in glycemic outcomes from baseline to week 12.

Outcome	Baseline	Week 12	*p*-Value	Mean Difference (95% CI)
TIR (70–180 mg/dL), %	62.53 ± 14.93	72.97 ± 10.88	<0.001	+10.43 (+7.07 to +13.80)
TAR (181–250 mg/dL), %	24.67 ± 11.01	19.37 ± 8.25	0.002	−5.30 (−8.52 to −2.08)
TAR (>250 mg/dL), %	8.90 ± 7.63	4.63 ± 4.19	<0.001	−4.27 (−6.00 to −2.53)
TBR (54–69 mg/dL), %	3.53 ± 3.72	2.67 ± 2.34	0.133	−0.87 (−1.78 to +0.05)
TBR (<54 mg/dL), %	0.47 ± 0.73	0.40 ± 0.50	0.564	−0.07 (−0.31 to +0.17)
Mean sensor glucose, mg/dL	159.30 ± 26.10	147.87 ± 17.05	<0.001	−11.43 (−16.86 to −6.01)
CV, %	35.66 ± 4.54	33.56 ± 4.49	0.029	−2.09 (−3.96 to −0.22)
GMI, %	7.10 ± 0.63	6.85 ± 0.40	0.001	−0.26 (−0.40 to −0.11)
Laboratory HbA1c, %	7.71 ± 1.17	7.08 ± 0.57	<0.001	−0.63 (−1.03 to −0.23)
Laboratory HbA1c, mmol/mol	61 ± 13	54 ± 6	—	−6.9 (−11.3 to −2.6)

Data are presented as means ± SDs. *p-*values are from paired-samples *t*-tests for TAR 181–250 mg/dL, mean sensor glucose, CV, and GMI, and Wilcoxon signed-rank tests for TIR, TAR >250 mg/dL, TBR, and laboratory HbA1c. Mean differences are week 12 minus baseline, with 95% CIs based on the t-distribution of the paired differences. TIR is the primary outcome; all other outcomes are secondary. HbA1c is reported in NGSP units (%) and IFCC units (mmol/mol); mmol/mol values are converted from HbA1c (%). GMI was read from each participant’s sensor report rather than calculated from mean sensor glucose, which is why the change in GMI is not an exact linear transformation of the change in mean sensor glucose; see Section 3.2. For outcomes compared with the Wilcoxon signed-rank test, Hodges–Lehmann median differences with distribution-free confidence intervals are given in Supplementary Table S3, and both paired *t*-test and Wilcoxon *p*-values for every outcome in Supplementary Table S6. CI, confidence interval; CV, coefficient of variation; GMI, glucose management indicator; TAR, time above range; TBR, time below range; TIR, time in range; SD, standard deviation.

**Table 3:** Changes in anthropometric measures, insulin doses, and dietary intake from baseline to week 12.

Outcome	Baseline	Week 12	*p*-Value	Test
Body weight, kg	68.73 ± 14.93	68.89 ± 14.33	0.741	t
BMI, kg/m^2^	24.22 ± 4.41	24.25 ± 4.04	0.854	t
Total daily insulin dose, U/day	41.53 ± 13.15	39.53 ± 14.15	0.115	W
Basal insulin dose, U/day	20.83 ± 8.10	20.40 ± 8.22	0.289	W
Bolus insulin dose, U/day	20.70 ± 6.56	19.13 ± 7.89	0.102	W
Energy intake, kcal/day	1629.38 ± 414.56	1591.37 ± 378.20	0.432	t
Carbohydrate intake, g/day	156.00 ± 54.57	153.32 ± 51.21	0.598	t
Carbohydrate, % energy	39.07 ± 6.29	39.61 ± 7.21	0.647	t
Protein intake, g/day	71.09 ± 17.37	71.11 ± 18.40	0.994	t
Fat intake, g/day	77.78 ± 20.12	74.58 ± 19.08	0.383	t
Fiber intake, g/day	22.84 ± 5.44	23.93 ± 6.97	0.324	t

Data are presented as means ± SDs. *p*-values are from paired-samples *t*-tests (t) or Wilcoxon signed-rank tests (W), as indicated in the Test column; all comparisons in this table are exploratory. Dietary intake was assessed from 3-day food records. BMI, body mass index; SD, standard deviation.

## Data Availability

The de-identified participant-level dataset analyzed in this study is provided as Supplementary Data S1, and the analysis syntax that reproduces every value reported in Table 1, Table 2 and Table 3 and Supplementary Tables S1–S8 from that dataset is provided as Supplementary File S1. The dataset contains no identifying information; sharing of de-identified data is covered by the ethics approval and the participants’ informed consent.

## Supplementary Materials

The following supporting information can be downloaded at https://www.mdpi.com/article/10.3390/nu18213640/s1, Table S1: Individualized insulin-to-carbohydrate ratios, insulin sensitivity factor, and formula-based estimates; Table S2: Sensitivity analysis of glycemic outcomes restricted to participants meeting the prespecified baseline BMI eligibility criterion (*n* = 22); Table S3: Medians and interquartile ranges for the outcomes compared with the Wilcoxon signed-rank test; Table S4: Findings from the 22-item baseline questionnaire (n = 30); Table S5: Sensitivity analysis of glycemic outcomes excluding the participant with a baseline laboratory HbA1c of 12.3% (n = 29); Table S6: Paired *t*-test and Wilcoxon signed-rank *p-*values for every outcome (n = 30); Table S7: Reported macronutrient distribution compared with the Turkey Dietary Guidelines 2022 (n = 30); Table S8: CGM system by insulin delivery mode and glycemic outcomes by CGM system; Data S1: de-identified participant-level dataset; File S1: analysis syntax.

## Abbreviations

BeBiS	Nutrition Information System
BMI	body mass index
CCE	carbohydrate-counting education
CGM	continuous glucose monitoring
CI	confidence interval
CSII	continuous subcutaneous insulin infusion
CV	coefficient of variation
GMI	glucose management indicator
HbA1c	glycated hemoglobin
HPLC	high-performance liquid chromatography
ICR	insulin-to-carbohydrate ratio
IFCC	International Federation of Clinical Chemistry and Laboratory Medicine
ISF	insulin sensitivity factor
MDI	multiple daily injections
NGSP	National Glycohemoglobin Standardization Program
SD	standard deviation
SPSS	Statistical Package for the Social Sciences
TAR	time above range
TBR	time below range
TIR	time in range.
